# Mesenchymal stromal/stem cells and bronchopulmonary dysplasia

**DOI:** 10.3389/fcell.2023.1247339

**Published:** 2023-10-30

**Authors:** Shuqing Zhang, Cassidy Mulder, Suzette Riddle, Rui Song, Dongmei Yue

**Affiliations:** ^1^ School of Pharmacy, China Medical University, Shenyang, China; ^2^ Liberty University College of Osteopathic Medicine, Lynchburg, VA, United States; ^3^ Cardiovascular Pulmonary Research Laboratories, Departments of Pediatrics and Medicine, University of Colorado Anschutz Medical Campus, Aurora, CO, United States; ^4^ Lawrence D. Longo, MD Center for Perinatal Biology, Department of Basic Sciences, Loma Linda University School of Medicine, Loma Linda, CA, United States; ^5^ Department of Pediatrics, Shengjing Hospital of China Medical University, Shenyang, Liaoning, China

**Keywords:** stem cells, bronchopulmonary dysplasia, development, extracellular vesicles, mitochondria

## Abstract

Bronchopulmonary dysplasia (BPD) is a common complication in preterm infants, leading to chronic respiratory disease. There has been an improvement in perinatal care, but many infants still suffer from impaired branching morphogenesis, alveolarization, and pulmonary capillary formation, causing lung function impairments and BPD. There is an increased risk of respiratory infections, pulmonary hypertension, and neurodevelopmental delays in infants with BPD, all of which can lead to long-term morbidity and mortality. Unfortunately, treatment options for Bronchopulmonary dysplasia are limited. A growing body of evidence indicates that mesenchymal stromal/stem cells (MSCs) can treat various lung diseases in regenerative medicine. MSCs are multipotent cells that can differentiate into multiple cell types, including lung cells, and possess immunomodulatory, anti-inflammatory, antioxidative stress, and regenerative properties. MSCs are regulated by mitochondrial function, as well as oxidant stress responses. Maintaining mitochondrial homeostasis will likely be key for MSCs to stimulate proper lung development and regeneration in Bronchopulmonary dysplasia. In recent years, MSCs have demonstrated promising results in treating and preventing bronchopulmonary dysplasia. Studies have shown that MSC therapy can reduce inflammation, mitochondrial impairment, lung injury, and fibrosis. In light of this, MSCs have emerged as a potential therapeutic option for treating Bronchopulmonary dysplasia. The article explores the role of MSCs in lung development and disease, summarizes MSC therapy’s effectiveness in treating Bronchopulmonary dysplasia, and delves into the mechanisms behind this treatment.

## 1 Introduction

Bronchopulmonary dysplasia (BPD) is a chronic lung disease of preterm infants characterized by impaired alveolar and vascular development, resulting in poor gas exchange and significant respiratory morbidity (Jobe, 2011; Gilfillan et al., 2021; Schmidt and Ramamoorthy, 2022). The primary drivers of BPD pathology are inflammation, oxidative stress, and parenchymal fibrosis (Higano et al., 2018; Benjamin et al., 2021; Holzfurtner et al., 2022; Kimble et al., 2022). BPD causes long-term issues like emphysema and pulmonary hypertension (Gough et al., 2014; Vom Hove et al., 2014).

MSCs have emerged as a promising candidate for BPD therapy due to their capacity for self-renewal, high proliferative potential, and ability to differentiate into cell types of mesodermal lineage (Thebaud et al., 2021). They also have immunomodulatory properties that allow allogeneic transplantation. Several clinical trials have evaluated MSC therapy in BPD patients. Intratracheal or intravenous MSC administration has decreased pro-inflammatory cytokines in tracheal aspirates, improved alveolarization, and reduced BPD severity (Alvarez-Fuente et al., 2018). While initially, it was thought that MSCs engrafted and differentiated directly into damaged lung tissue, recent evidence indicates paracrine signaling is the primary mechanism of action. Several recent studies have shown that MSCs communicate with other cells through paracrine signaling (Di Bernardo et al., 2014; Obendorf et al., 2020). MSC paracrine signaling of anti-apoptotic, anti-inflammatory, and pro-angiogenic bioactive substances to the microenvironment through extracellular vesicles (EVs) is thought to be the primary means of communication between MSCs and injured lung tissue (Lai et al., 2010; Aliotta et al., 2016; Reiter et al., 2017; Varkouhi et al., 2019; Varderidou-Minasian and Lorenowicz, 2020; Hao et al., 2022; Xiong et al., 2023). In particular, MSC-derived EVs like exosomes and endosomes have emerged as critical intercellular communicators that can reprogram damaged cells through the transfer of bioactive cargo (Jadli et al., 2020). MSC-EVs have been found to enhance cell proliferation, inhibit apoptosis, stimulate cell migration, modulate differentiation, promote angiogenesis, regulate immune responses, suppress inflammation, improve mitochondrial function, and attenuate oxidative stress. By orchestrating these diverse restorative effects in the damaged neonatal lung, MSC-EV therapies may alleviate structural and vascular defects associated with arrested alveolarization and dysregulated vascular development in BPD (Mansouri et al., 2019; Abele et al., 2022; Sharma et al., 2022).

The mitochondria are essential to the normal development and function of lung cells, including the regulation of metabolism, growth, differentiation, and injury responses (Zhang Y. et al., 2022). Bronchopulmonary dysplasia is associated with defects in mitochondrial structure, dynamics, DNA integrity, and oxidative metabolism (Xuefei et al., 2021). MSC therapy has been shown to alleviate mitochondrial dysfunction in BPD animal models, leading to improved alveolar and lung vascular growth (Islam et al., 2012; Dutra Silva et al., 2021). There is emerging evidence that mesenchymal stem cells (MSCs) can directly donate healthy mitochondria to damaged lung cells to restore mitochondrial function (Willis et al., 2018). In addition, MSCs could stimulate mitochondrial replication in injured lung cells through paracrine signaling molecules and extracellular vesicles (Phinney et al., 2015; Willis et al., 2018). Thus, MSCs may help resolve detrimental inflammation that can further impair mitochondrial and cellular function in lung cells injured by disease. By understanding how MSCs transfer mitochondria, promote mitochondrial biogenesis, and reduce mitochondrial inflammation, new therapeutic approaches may be developed to treat respiratory diseases and injuries caused by mitochondrial dysfunction.

This article explores the multifaceted role that MSCs play in lung development and disease. Additionally, we will provide an overview of current knowledge on the effectiveness of MSC therapy in treating BPD and shed light on the mechanisms driving this approach.

## 2 The role of mesenchymal stem cells in mammalian lung development

Mammalian lung development is precisely controlled by dynamic epithelial-mesenchymal interactions (Morrisey and Hogan, 2010). By controlling epithelial differentiation and function, MSCs play an essential role in lung morphogenesis and homeostasis (Driscoll et al., 2012). Through paracrine signaling, MSCs also secrete growth factors that influence lung development.

### 2.1 Lung MSC origins and characteristics

During embryogenesis, lung MSCs originate from the splanchnic mesoderm and undergo proliferation and differentiation into specialized mesenchymal cells (Cardoso and Lu, 2006). These lung MSCs exhibit clonogenicity, self-renewal, and multidifferentiation potential into mesodermal lineages (Jarvinen et al., 2008). Several studies have highlighted the intimate relationship between MSCs and their mitochondria. A healthy and efficient mitochondrial system is critical for optimizing MSCs (Forni et al., 2016). MSCs undergo rapid proliferation and differentiation during lung development, requiring mitochondrial energy. As differentiation proceeds, mitochondria undergo a metabolic shift toward OXPHOS, indicating their essential role in MSC maturation (Pattappa et al., 2011). For instance, during the differentiation of bone marrow mesenchymal stromal cells (BMSCs), mitochondrial length increased along with decreased DLP1 protein levels and increased OPA1 protein levels; this indicates a shift toward mitochondrial fusion, which maintains BMSC stemness (Feng et al., 2019). MSC differentiation can be hampered by dysfunctional mitochondria, characterized by decreased membrane potential, impaired OXPHOS, and increased ROS (Chen et al., 2008). Another fascinating aspect is MSCs’ ability to transfer mitochondria. As a result of mitochondrial transfer from MSCs to damaged cells, the recipient cells are more likely to be repaired and recovered (Islam et al., 2012). The mechanism may be instrumental in repairing lung injuries and preserving lung health.

### 2.2 MSCs in lung branching morphogenesis

In the surrounding mesenchyme of the bronchi, mesenchymal progenitors contribute to the differentiation of smooth muscle and endothelial cells. Mitochondrial capacity and ATP production in the mesenchyme were disrupted, resulting in MSC absence and impaired airway branching (Zhang K. et al., 2022). Conducting airways are formed by dichotomous branching morphogenesis triggered by inductive signals from the distal lung mesenchyme, such as fibroblast growth factor 10 (FGF10) (Bellusci et al., 1997). MSCs provide key signals regulating this process. Deletion of β-catenin in lung MSCs disrupted airway branching (De Langhe et al., 2008). Also, Wnt signaling in MSCs regulated FGF10 expression and epithelial branching (Shu et al., 2005). Sonic hedgehog (SHH) signaling in MSCs similarly regulates airway patterning (Ikonomou et al., 2020). Intriguingly, disrupting mitochondrial dynamics in the mesenchyme impaired airway branching (Liu et al., 2021). Crosstalk between mitochondrial networks and other cell signaling pathways that regulate MSC growth, migration, and paracrine signaling during branching morphogenesis is of particular interest.

### 2.3 MSCs in alveolar development and regeneration

Mesenchymal cells secrete VEGF, TGF-β, and FGFs, influencing alveolar differentiation, proliferation, and formation. The ablation of FGF10 from MSCs impaired alveolar formation (Volckaert et al., 2013). Paracrine signals such as SHH and VEGF regulate MSC differentiation of alveolar epithelial cells (Liu J. et al., 2022). Depleting lung MSCs post-pneumonectomy severely compromised compensatory alveolar regeneration (Zhen et al., 2008). A key role in alveolar formation is played by mesenchymal mitochondrial activity and distribution regulated by mTORC1 pathway. Therefore, MSCs are essential for alveolar development and regeneration after injury. Much is still to be learned about how modulating mitochondrial biogenesis, dynamics, and mitophagy in lung MSCs impacts alveolar epithelial cell growth, differentiation, and matrix production during alveolarization.

### 2.4 MSCs in vascular development

The mesenchyme layer and cells exhibit high VEGF, stimulating hemangioblasts to form blood pools (Greenberg et al., 2002). Deleting β-catenin in lung MSCs disrupted vascular patterning (De Langhe et al., 2008). Consequently, MSCs provide the trophic signals necessary for coordinated lung vascular development.

**FIGURE 1:**
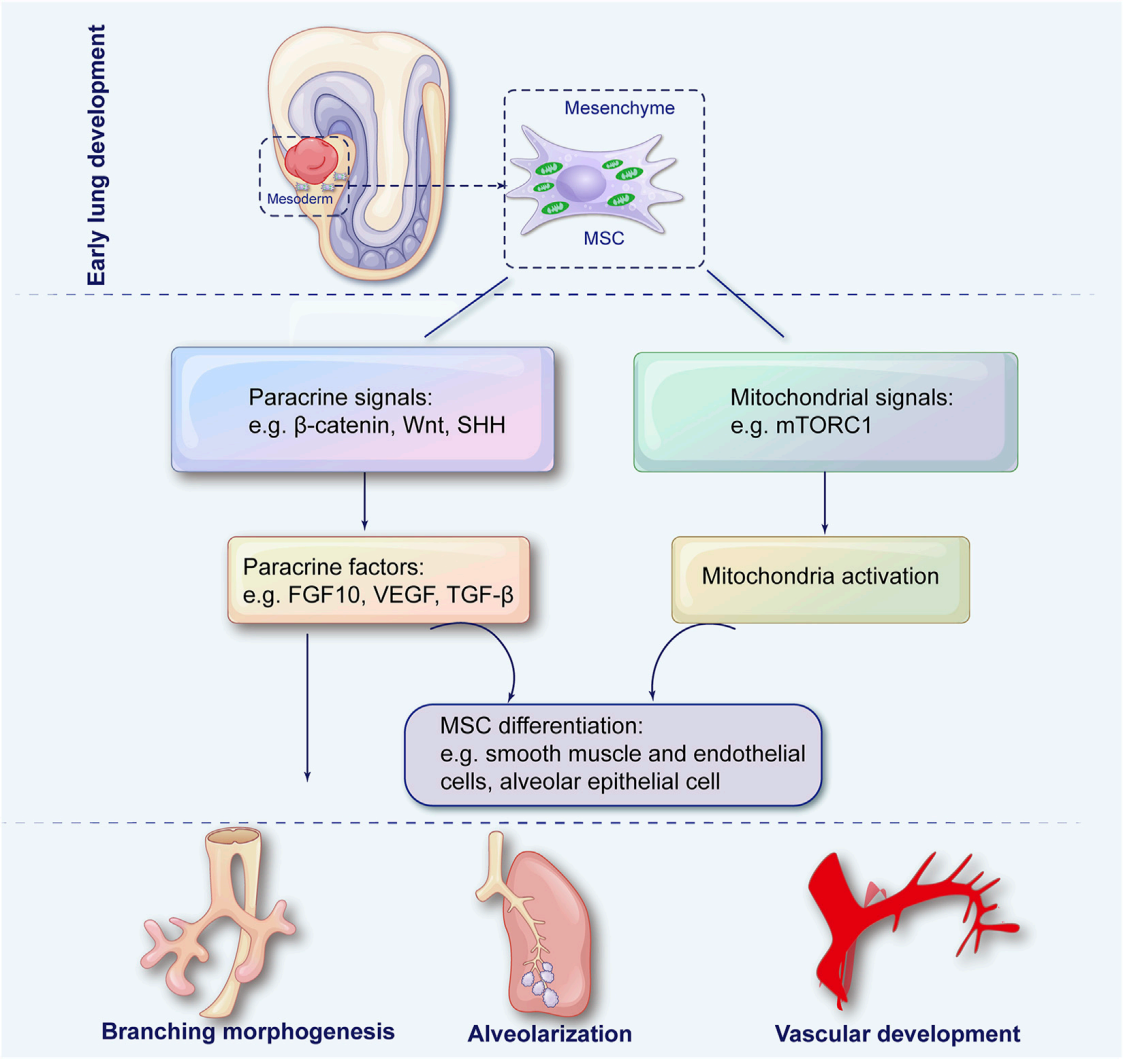
The role of mesenchymal stem cells in pulmonary development. During embryonic development, lung mesenchymal stem cells (MSCs) arise from the splanchnic mesoderm and subsequently differentiate into specialized mesenchymal lineages through paracrine signaling and mitochondrial regulation to coordinate key processes in lung morphogenesis, including airway branching, alveolarization, and vascular development.

As a result, lung MSCs are crucial for airway branching, alveolarization, and vascular development throughout lung morphogenesis. Lung development is orchestrated by the intricate dance between mitochondria and MSCs (Figure 1). Despite our growing understanding over the years, much remains to be explored. Future studies elucidating the molecular and cellular mechanisms underlying their interaction can pave the way for innovative therapeutic strategies in lung medicine.

## 3 Interaction between mesenchymal stem cells and bronchopulmonary dysplasia

Several prenatal risk factors for BPD have been identified, including maternal smoking, hypertension, placental insufficiency, and chorioamnionitis (Klinger et al., 2013; Mizikova and Thebaud, 2023). Placental abnormalities leading to imbalanced pro- and anti-angiogenic signaling may disrupt fetal lung vascular development, significantly impacting BPD susceptibility and severity (Hansen et al., 2010; Ozkan et al., 2012; Torchin et al., 2016; Sucre et al., 2021). Premature infants born during the canalicular and saccular stages of lung development are at highest risk for developing BPD (Jobe, 2011). Endogenous MSCs play a critical role in alveolarization through the tightly regulated processes of alveolar septation and vascularization (Morrisey and Hogan, 2010; Klein, 2021). The decrease or functional impairment in resident stem cells within the lung tissue of infants leads to simplified alveolar architecture and abnormal pulmonary vasculature contributing to BPD (Alphonse et al., 2012; Collins and Thebaud, 2014; Simones et al., 2018).

Extensive research has been conducted on how interference with MSCs and their signaling pathways can result in the onset of BPD. MSCs, similar to fibroblast cells, are found in the mesodermal tissue in adults and fetuses (Anker et al., 2003; Fang et al., 2019). Their presence has been noted in a variety of locations including bone marrow, adipose tissue, lung tissue, the placenta, and the stroma of the umbilical cord (Anker et al., 2003; Igura et al., 2004; Nguyen et al., 2022). BPD treatment methods such as hyperoxia and mechanical ventilation can negatively impact the functionality of the MSCs residing in the lung. Interestingly, these cells are copious in fetal lungs and can be a biomarker for predicting BPD onset when retrieved from tracheal aspirates of prematurely born infants. Growth factor expression involved in alveolar development and repair, such as VEGF and FGF10, is reduced in these MSCs (Mobius et al., 2019).

The precise mechanisms through which endogenous MSCs switch from protecting against BPD to contributing to BPD remain incompletely understood. The MSCs can repair damaged cells by promoting growth and differentiation. For instance, a subset of Dermo1+ endogenous MSCs has been recognized as having the capacity to function as stem cells in regenerating airway epithelial cells during the repair process following LPS-induced acute lung injury (Fang et al., 2019). Current data indicates that paracrine signaling and immune modulation are likely key players in mitigating hyperoxia-induced lung injury. EVs often facilitate paracrine signaling, carrying growth factors such as TGF-β. In a BPD model triggered by hyperoxia, an escalation in TGF-β expression was observed in the lungs, leading to structural changes; this included the multiplication of α-actin-positive myofibroblasts within the alveolar septal wall and the emergence of abnormal alveolar and vascular structures (Luan et al., 2015). Interestingly, when bone marrow stem cells were administered, a considerable decline in TGF-β levels was observed (Luan et al., 2015). These outcomes suggest that TGF-β could stimulate abnormal differentiation of alveolar mesenchymal progenitor cells into myofibroblasts, eventually playing a role in BPD progression. Furthermore, under intrauterine circumstances, human fetal lung MSCs were observed to generate considerable quantities of elastin and sulfated glycosaminoglycans, key constituents required for typical lung development. Examination of the secretome revealed that human fetal lung MSCs could be responsible for secreting molecules essential for angiogenic and inflammatory signaling, such as IL-8, VEGF, angiogenin and angiopoietin-1 (Mobius et al., 2019). Secretome analysis also revealed the existence of molecules that promote epithelial cell maturation and protection, such as keratinocyte growth factor/FGF7, FGF10 and the antioxidant known as stanniocalcin-1. In addition, inflammatory mediators such as monocyte chemotactic protein-1, stromal cell–derived factor-1, IL-6, and the tissue inhibitor of metalloproteinases 1 were also detected (Mobius et al., 2019). Exposing lung MSCs to hyperoxia recapitulated some harmful events associated with post-premature birth, such as cell apoptosis, decreased colony-forming capabilities, and alterations in MSCs’ surface markers. It hindered the secretion of factors crucial for lung growth. These effects may play a part in the development of BPD (Mobius et al., 2019).

In the following section, we delve into the regulatory role of MSCs in the complex pathogenesis of BPD (Table 1), intending to identify potential targets for MSC-based BPD treatments.

**TABLE 1:** Effects of decreased and dysfunctional endogenous mesenchymal stem cells on BPD in preclinical experiments.

	Species	Pathophysiological factor	Signaling	Altered MSCs	Injured lung	References
*In vitro*	Human	oxidative stress/mitochondrial dysfunction	PINK1 stanniocalcin-1	↓ electron transport chain complex-IV activity, basal and maximal oxygen consumption rates, spare respiratory capacity, and ATP-linked OCR	disruption of lung development	Mobius et al. (2019); Hazra et al. (2022)
				↑ proton leak		
				↓ mitophagy ↓MSC number		
	Human	aberrant angiogenesis	SOX-2 and OCT-4	↓ MSC number ↓expression of VEGF, FGF-10, and angiogenin	↓ alveolar development and repair ↓lung vascularization	Mobius et al. (2019)
	Human	aberrant angiogenesis	SHH	↓ expression of proangiogenic genes FGF-9 and IL-6	↓ capillary density	White et al. (2007); Kwon et al. (2014)
	Human	Inflammation	FoxF1, Wnt5a, and Tbx3	↑ release of pro-inflammatory cytokines CXCL1, IL-6, and IL-8	↑ inflammation ↑ fibrosis	Bozyk et al. (2011)
				↓ CXCL1/GRO-α and HGF		
	Human	Inflammation	NFκB	↑ Pro-inflammatory cytokines TNF-α, IL-1β, IL-6, and IL-8	↑ inflammation	Reicherzer et al. (2018)
				↓MSCs number		
*In vivo*	Rat	aberrant angiogenesis	Semaphorins 3A and 3E	↓ expression of FGF10	↓ capillary density	Collins et al. (2018)
	Mouse	Inflammation	TGF-β	↑ MSCs apoptosis ↓MSCs number	↑ monocytes/macrophages and neutrophils	Ehrhardt et al. (2016)
					↑IL-1β, CXCL1, and MCP-1 ↑ impaired alveolar structure	

BPD, bronchopulmonary dysplasia; MSCs, mesenchymal stromal/stem cells; PINK1, PTEN-induced putative kinase 1; ATP, adenosine triphosphate; OCR, oxygen consumption rate; SOX-2, SRY-box2; OCT-4, Octamer-binding transcription factor 4; VEGF, vascular endothelial growth factor; FGF, fibroblast growth factor; SHH, sonic hedgehog; IL, interleukin; Tbx3, T-box transcription factor3; CXCL1, C-X-C motif chemokine ligand 1; GRO-α, growth-related oncogene-α; HGF, hepatocyte growth factor; NFκB, Nuclear factor kappa B, TNF-α, tumor necrosis factor-α; TGF-β, transforming growth factor-β; MCP-1, monocyte chemoattractant protein-1.

### 3.1 Oxidative stress

While fetal lungs usually develop in a hypoxic intrauterine environment, BPD is a condition precipitated by premature exposure to hyperoxic conditions at birth, due either to oxygen treatment in efforts to oxygenate the infants sufficiently or simply by exposure to the relative hyperoxic conditions of the atmosphere. This condition subjects infants with extremely low birth weights to oxidative stress (Kimble et al., 2022). Cellular components such as the mitochondria: its DNA, the electron transport chain, and reactive oxygen generation machinery are particularly susceptible to oxidant damage. The function of mitochondria is pivotal in moderating the response to oxidative stress and affects the pluripotency and regenerative potential of MSCs (Paliwal et al., 2018). In a study exploring the correlation between mitochondrial function and the MSC function, electron transport chain complex-IV activity, basal and maximal oxygen consumption rates, spare respiratory capacity, and ATP-linked oxygen consumption rate were reduced. Proton leak was increased in MSCs from extremely low birth weight infants who either perished or developed moderate/severe BPD, compared to MSCs from infants who survived with none to mild BPD (Hazra et al., 2022).

PTEN-induced putative kinase 1 (PINK1) mediated mitophagy is one mechanism cells use to eliminate dysfunctional mitochondria. As a result, oxidative stress-induced mitochondrial dysfunction could lead to MSC depletion, thus obstructing lung development. Another finding described that MSCs from infants who died or developed moderate/severe BPD after O2 treatment showed lower PINK1 expression, implying decreased mitophagy (Hazra et al., 2022). The antioxidant stanniocalcin-1 was also reduced in fetal lung MSCs exposed to hyperoxia (Mobius et al., 2019).

In conclusion, prenatal risk factors for BPD, such as hyperoxic conditions, lead to oxidative stress and MSC dysfunction, which can contribute to BPD’s development and advancement.

### 3.2 Angiogenesis

Pulmonary microvascular dysplasia is a component of BPD pathogenesis; therefore, fostering pulmonary angiogenesis and enhancing vasculogenesis are crucial objectives to mitigate this dysplasia. Under hyperoxic conditions, it was found that MSCs in the tracheal aspirates of preterm infants’ lungs exhibited reduced expression of growth factors crucial for alveolar development and repair, such as VEGF, FGF10, and angiogenin, through the decrease in SOX-2 and OCT-4 (Mobius et al., 2019). Moreover, the CD146+ MSCs in the lungs exposed to hyperoxia displayed decreased gene expression of notable proangiogenic genes, including FGF9 and IL-6, through SHH signaling (White et al., 2007; Kwon et al., 2014). In a rat model of BPD induced by hyperoxia, the resident lung CD146+ MSCs were found to inhibit rather than promote angiogenesis through the axon guidance signaling (Semaphorins 3A and 3E) (Collins et al., 2018). Consequently, BPD insults prompt alterations in angiogenic pathways, which result in an MSC phenotype with aberrant microvascular development, a key characteristic of BPD pathogenesis (Alvira, 2016).

An investigation involving hyperoxia-induced BPD in rodent models reported decreased endothelial progenitor cells within the bloodstream, lungs, and bone marrow (Balasubramaniam et al., 2007). Endothelial progenitor cells, also called endothelial colony-forming cells, are required for vasculogenesis in the developing lung and are associated with the support and maturation of endothelial progenitor cells. Crucial to the regional specialization of embryonic lung tissue, MSCs at the outer edge of branching epithelium are noted for producing FGF10, a vital element in the signaling interplay involving BMP, Wnt, and sonic hedgehog pathways that direct the differentiation of epithelial stem/progenitors during lung development (Morrisey and Hogan, 2010). *In vitro* studies have shown that MSCs substantially support the growth and differentiation of epithelial stem cells (McQualter et al., 2010). Further highlighting this critical microenvironmental association, recent *in vivo* research employing the naphthalene injury model showed that parabronchial mesenchymal cells discharge FGF10 to stimulate epithelial regeneration in the remaining progenitor cells (McQualter et al., 2010). These findings support the theory that a reduction in MSCs or malfunctioning MSCs might cause the hindered growth of endothelial progenitor cells and affect their angiogenic supportive capacity, thereby contributing to the onset of BPD.

### 3.3 Inflammation

Inflammatory imbalance plays a significant role in the development of BPD. Various immune regulatory attributes have been credited to MSCs from different tissue origins, contributing to tissue regeneration, cell death prevention, tissue fibrosis inhibition, and the reduction of tissue damage (Li et al., 2019). MSCs isolated from the tracheal aspirates of preterm infants show a propensity towards inflammation, secreting increased levels of pro-inflammatory cytokines like CXCL-1, IL-6, and IL-8 and decreased anti-fibrotic factor levels of CXCL1/GRO-α and HGF, possibly through dysregulated FoxF1, Wnt5a, and Tbx3 signaling (Bozyk et al., 2011). Traditional pro-inflammatory cytokines such as TNF-α, IL-1β, IL-6, and IL-8 trigger comparable changes in MSCs derived from preterm infants with severe BPD. NFκB targeting was observed to reverse this pro-inflammatory tendency (Reicherzer et al., 2018). In the lungs of infants suffering from BPD, it was discovered that thicker alveolar walls were linked with a scarcity of PDGFR-α-positive cells in the malformed alveolar septa (Popova et al., 2014). A significant decrease in the volume of PDGFR-α-positive alveolar tips was observed in the lungs of neonatal mice exposed to excess oxygen in another study (Popova et al., 2014). Another study demonstrated enhanced apoptosis in PDGFR-α-positive mesenchymal cells through abrogation of TNF-α-mediated NFκB signaling and primarily influenced by TGF-β1 signaling (Ehrhardt et al., 2016). The authors concluded that this phenomenon explained much of the amplified lung damage during mechanical ventilation (Ehrhardt et al., 2016).

Alveolar macrophages, responsible for maintaining a balanced immune response in the lung, react to changing environmental conditions in various ways, often simplified into anti-inflammatory and pro-inflammatory effects. Hyperoxia, a prevalent condition in BPD supportive care, can damage alveolar macrophages. PGE2, a substance secreted by MSCs, plays a pivotal role in the bioenergetic shift in macrophages, increasing anti-inflammatory activation and decreasing pro-inflammatory activation through AMPK activity/SIRTUIN 1 signaling (Vasandan et al., 2016). Levels of prostaglandin E2 were found to be reduced in hyperoxia–exposed fetal lung MSCs (Mobius et al., 2019). Consequently, the decrease in anti-inflammatory MSCs or the impairment of their anti-inflammatory function contributes to the pathogenesis of BPD.

## 4 Stem cell-based BPD preclinical treatments

### 4.1 *In vivo* studies

**FIGURE 2:**
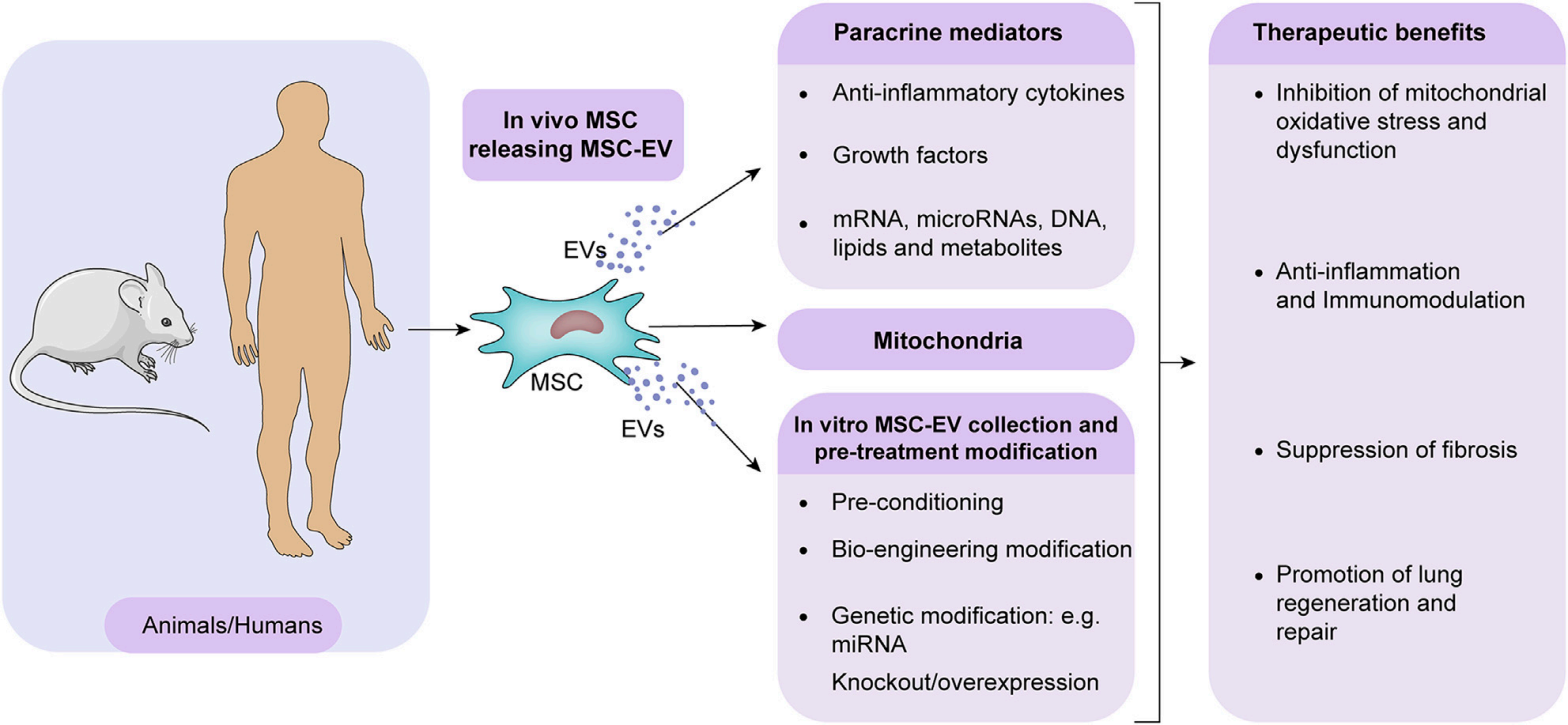
MSC-EVs mediate therapeutic effects in BPD. MSCs predominantly promote lung repair in BPD through paracrine. MSCs release EVs containing bioactive molecules and mitochondria that mitigate injury. In addition, EV isolation offers the potential for cell-free treatment. MSC-EVs are enhanced in their regenerative capacity by pre-conditioning, bioengineering, and genetic modification *in vitro*. Optimizing EV production and delivery to maximize lung localization may enable clinical translation for treating BPD.

Studies using animal models continue to refine the use of MSCs in therapeutic strategies. The potential of MSCs to restore normal lung function has sparked a desire to understand their interplay with damaged lung tissue. As discussed, MSCs act through paracrine signaling, with EVs playing an essential role in this mechanism (Figure 2). The EVs are between 30 and 1000 nm in size and are secreted by most cell types. After binding and internalization, they initiate signal transduction in target cells (Omar et al., 2022). Typically, they arise from multivesicular bodies whose plasma membranes are budding inward. The EVs contain cytokines, proteins, mRNA, microRNAs, DNA, lipids, metabolites and even mitochondria from the parent cells. Several mechanisms can enable EVs to transfer their molecular cargo when released, including fusing with target cell membranes or being endocytosed (Jadli et al., 2020). EVs can modulate recipient cells, affecting immunity, development, homeostasis, and disease pathogenesis. Preconditioning MSCs *in vitro* can enhance the regenerative capacity of secreted EVs. MSC-EVs can also be enhanced therapeutically by bioengineering or genetic modification (Figure 2). By optimizing EV production protocols and delivery strategies, MSC-EV therapy for BPD may be translated into clinical practice. A better understanding of MSC-EV cargo and underlying mechanisms of lung repair will facilitate the development of more potent acellular treatments. Several studies have found that MSC-EVs have protective effects on the lung by blocking inflammation, improving lung function, and reducing pulmonary hypertension (Mansouri et al., 2019; Abele et al., 2022; Sharma et al., 2022). A recent study discovered that MSCs release several growth factors linked to angiogenesis, predominantly VEGF, stored in EVs, particularly exosomes (Xi et al., 2022); this suggests a potential method for MSC-exosomes to increase VEGF-driven angiogenesis in BPD treatment (Xi et al., 2022). An experimental study evaluating a hyperoxia-induced BPD rat model compared the protective effects of intratracheally administered MSCs *versus* EVs to ascertain the differences between MSC and MSC-derived extracellular vesicle administration. The study found that both MSCs and MSC-EVs alleviated hyperoxia-induced damage, but MSC-EVs achieved superior outcomes regarding alveolarization and lung vascularization (Braun et al., 2018; Moreira et al., 2020). The benefits of using EVs over MSCs for treatment include easier intratracheal administration, the potential for modifications to enhance the benefits of extracellular vesicle treatment, and the option to freeze-dry (lyophilize) them to preserve their biological activity.

As highlighted earlier, decreased VEGF results in compromised angiogenesis, significantly contributing to BPD development. Extended hyperoxia interrupts normal lung development, a phenomenon linked with abnormal expression of Akt and VEGF (You et al., 2020). A study on a hyperoxia-induced rat model of BPD found that the expressions of phosphorylated Akt and VEGF-A significantly increased following treatment with small EVs derived from human umbilical cord mesenchymal stem cells. At the same time, PTEN and cleaved caspase-3 expression were found to negatively correlate with phosphorylated AKT in the lungs after treatment with these small EVs (You et al., 2020). PTEN functions as the primary regulator of Akt, and it has been documented that the signaling pathway involving PTEN and Akt governs cellular growth, programmed cell death (apoptosis), and the formation of new blood vessels (angiogenesis). Therefore, these findings suggest that small EVs, derived from human umbilical cord mesenchymal stem cells, boost pulmonary alveolarization and angiogenesis to counteract BPD through suppressing PTEN and activating Akt/VEGF pathway.

Research into the paracrine signaling of MSCs via EVs has been paralleled by studies delving into microRNA. One study examined the effect of bone marrow-derived MSCs on microRNA (miR) in a trial involving newborn mice. MiRs, non-coding RNAs of 21–25 nucleotides in length, have roles in several biological processes, including cell proliferation, cell death, resistance to stress, and tumorigenesis. MiR-206 is known to be downregulated in both patients with BPD and newborn mice. Previous research has highlighted the role of miR-206 in BPD by demonstrating its impact on the expression of fibronectin 1. This glycoprotein, a vital component of the extracellular matrix (ECM), plays a crucial role in various biological processes such as cell adhesion and migration, embryonic development, wound repair, cancer metastasis, and immune responses (Zhang et al., 2013). Bone marrow-derived MSCs, when transfected with miR-206, showed functional regulation by enhancing the expression of surfactant protein C and reducing the levels of inflammatory cytokines such as fibronectin 1, TGF-β1, and IL-6. These changes led to a reduction in lung fluid accumulation (pulmonary edema) and an improvement in the formation of alveoli. This finding hints at the potential therapeutic benefits these MSCs might offer in treating BPD (Zhang et al., 2013).

Porzionato et al. (Porzionato et al., 2021) evaluated the protective effects of MSC-derived EVs administered intratracheally using a newborn rat model exposed to hyperoxia for 2 weeks and 4 weeks of recovery. The rats exposed to hyperoxia displayed decreased and expanded distal air spaces, along with sporadic areas of interstitial thickening. The study showed that the treatment with MSC-derived EVs prevented the accumulation of connective or fibrous tissue in the lung parenchyma and sustained the presence of CD163-positive macrophages in hyperoxia-exposed rats, compared to those maintained in normoxic conditions (Porzionato et al., 2021). The treatment did not decrease the total macrophage population but influenced the balance between pro-inflammatory and anti-inflammatory macrophages. Anti-inflammatory macrophages, identified by CD163, have been shown to facilitate alveolarization during normal lung development. The study disclosed that extracellular vesicle treatment averted a decline in the density of interstitial alveolar CD163+ macrophages in rats exposed to hyperoxia (Porzionato et al., 2021). Despite the theoretical benefit of preventing a decrease in CD163+ cells through extracellular vesicle treatment, ongoing damage might still cause these macrophages to stimulate excessive ECM deposition by activating resident fibroblasts via the release of pro-fibrotic factors (Porzionato et al., 2021); this implies additional mechanisms play a part in the anti-fibrotic effect of MSC-EVs, necessitating further research.

A recent study shows that MSC-derived extracellular vesicles can reshape the lung macrophage profile, reducing inflammation and immune reactions to counteract BPD caused by hyperoxia (Willis et al., 2018). The role of MSCs extends beyond paracrine processes (such as carriers of 150-nm exosomes). Alternatively, more giant vesicles (over 500 nm wide) or direct connections between cells might carry more intricate structures, such as mitochondria (Willis et al., 2018). Mitochondria’s potential role in MSC action could pave the way for more refined, effective MSC therapies for BPD.

### 4.2 *In vitro* studies

The study of placental mesenchymal stem cells (PMSCs) has grown in recent years due to their application in various physiological and pathological conditions. Numerous benefits can be gained from PMSCs, including their multipotency and immunomodulatory capabilities. In contrast to embryonic stem cells, PMSCs can be harvested from young donors in large quantities, overcoming ethical concerns associated with embryonic stem cells (Mathew et al., 2020; Zhang Y. et al., 2022). In an ex-vivo fetal lung culture model, PMSCs exert more effective stimulation on perinatal lung morphogenesis than BMSCs (Di Bernardo et al., 2014); this suggests PMSCs are excellent candidates for regenerative medicine, enabling their extensive use in various stem cell therapy studies. PMSCs have shown the capacity to preserve expanded umbilical cord blood CD34^+^ cells during cryopreservation, enhancing the recovery, survival, and functionality of these cells upon revival; this is achieved through the reduction of oxidative stress. Interestingly, PMSCs have outperformed cord MSCs in providing cryoprotection to the umbilical cord blood cells (Kadekar et al., 2016). A recent study found that co-cultivation with PMSCs boosted ATP production in trophoblasts by regulating calcium channel expression and inducing mild oxidative stress (Seok et al., 2020). Moreover, exosomes derived from human placenta choriodecidual membrane-derived mesenchymal stem cells significantly mitigated LPS-induced lipid peroxidation and apoptosis in the lung (Seok et al., 2020). These studies suggest that PMSC may help treat the cellular metabolic alterations associated with BPD. However, further research is required to explore the regulatory role of PMSCs in oxidative stress and mitochondrial function in the context of BPD development and treatment. Mass augmenting mitochondrial function and electron transport chain complexes could contribute to the therapeutic efficacy of MSCs, a concept currently under evaluation in clinical trials.

Another provocative finding is that the conditioned medium from PMSCs has been found to contain both factors that stimulate and inhibit angiogenesis, aiding the enhancement of endothelial tube formation. It was observed that endothelial cells take in exosomes from PMSCs, which promotes the formation and migration of tubes and enhances the expression of genes linked to angiogenesis, leading to better angiogenesis *in vivo* (Komaki et al., 2017). Growing attention is being focused on the possible role of PMSCs in influencing angiogenesis in the progression and treatment of BPD. Notably, PMSCs display ease of propagation and superior immunoregulatory attributes, rendering them an appealing treatment option for diseases linked with inflammation in the future (Wu et al., 2017). Studies have shown that human PMSCs can counteract the polarization of pro-inflammatory macrophages and release pro-inflammatory cytokines triggered by LPS by modulating TLR4 expression and the NF-κB signaling pathway (Liu Y. et al., 2022). Therefore, in the landscape of BPD, immune cells may represent promising targets for therapeutic interventions employing PMSC-derived EVs.

## 5 Stem cell-based BPD clinical applications

Numerous preclinical studies have conducted comprehensive experimental studies, leading to a handful of clinical trials currently underway to examine the impact of MSC therapy (Table 2). A pioneering phase I clinical trial was conducted on nine preterm infants, with gestational ages ranging from 23 to 29 weeks, who needed mechanical ventilation within 5–14 days after birth (Ahn et al., 2017). These infants received an intratracheal delivery of either 10^7^ or 2 × 10^7^ MSCs derived from the umbilical cord. This group of infants did not experience any unfavorable events, thus affirming the safety of the MSC treatment administered intratracheally (Ahn et al., 2017). Following this, the same research team reported that the treated infants displayed no deficits in neurological, respiratory, or growth parameters even after 2 years (Ahn et al., 2017). A noteworthy aspect of this phase I clinical trial is its establishment of the safety of intratracheal MSC administration, opening doors for future clinical trials. A different clinical trial investigates the safety and effectiveness of allogeneic human umbilical cord-derived MSCs (hUC-MSCs) delivered intravenously. The dosage was incrementally increased to gather supportive data for the safety of intravenously delivered hUC-MSCs in treating patients with severe BPD. Importantly, this trial is the first to focus on the therapeutic effects of hUC-MSCs in children suffering from severe BPD rather than merely the preventative effects (Wu et al., 2020).

**TABLE 2:** Clinical trials targeting MSCs in BPD treatment.

Cell type	Clinical trials number	Phase	Study title	Status	Study model	Administration methods	Interventions
hMSCs	NCT03683953	I	The Treatment of Bronchopulmonary Dysplasia by Intratracheal Instillation of Mesenchymal Stem Cells	unknown	Parallel Assignment	intratracheal administration	25 million cells/kg
							Normal saline without hUCB-MSCs
hMSCs	NCT02443961	I	Mesenchymal Stem Cell Therapy for Bronchopulmonary Dysplasia in Preterm Babies	completed	Single Group	N/A	3 doses of 5 million MSC
hUC-MSCs	NCT04062136	I	Umbilical Cord Mesenchymal Stem Cells Transplantation in the Treatment of Bronchopulmonary Dysplasia	unknown	Single Group	intravenous infusion	1 million cells per body kg
hUC-MSCs	NCT03558334	I	Human Mesenchymal Stem Cells For Bronchopulmonary Dysplasia	unknown	Parallel Assignment	intravenous infusion	Dose A- 1 million cells per body kg; Dose B- 5 million cells per body kg
							No intravenous infusion of hUC-MSCs
hUC-MSCs	NCT03601416	II	Human Mesenchymal Stem Cells For Moderate and Severe Bronchopulmonary Dysplasia	unknown	Parallel Assignment	intravenous infusion	Dose A- 1 million cells per body kg; Dose B- 5 million cells per body kg
							No intravenous infusion of hUC-MSCs
hUC-MSCs	NCT03631420	I	Mesenchymal Stem Cells for Prevention of Bronchopulmonary	recruiting	Single Group	N/A	Cohort 1: 3 million cells/kg; Cohort 2 : 10 million cells/kg; Cohort 3 : 30 million cells/kg
hUC-MSCs	NCT03873506	I	Follow-Up Study of Mesenchymal Stem Cells for Bronchopulmonary Dysplasia	unknown	Single Group	intravenous infusion	Dose A- 1 million cells per body kg; Dose B- 5 million cells per body kg
hUC-MSCs	NCT03645525	I/II	Intratracheal Umbilical Cord-derived Mesenchymal Stem Cell for the Treatment of Bronchopulmonary Dysplasia (BPD)	recruiting	Parallel Assignment	Single intratracheal administration	2 × 10^7/kg per body kg
							Saline without hUC-MSCs
hUC-MSCs	NCT02381366	I/II	Safety and Efficacy of PNEUMOSTEM^®^ in Premature Infants at High Risk for Bronchopulmonary Dysplasia (BPD) - a US Study	completed	Single Group	N/A	Dose A: 10 million cells per kg; Dose B: 20 million cells per kg
hUC-MSCs	NCT03774537	I/II	Human Mesenchymal Stem Cells For Infants At High Risk For Bronchopulmonary Dysplasia	unknown	Parallel Assignment	intravenous infusion	Dose A- 1 million cells per kg; Dose B- 5 million cells per kg
							No intravenous infusion of hUC-MSCs
hUC-MSCs	NCT01207869	I	Intratracheal Umbilical Cord-derived Mesenchymal Stem Cells for Severe Bronchopulmonary Dysplasia	unknown	Parallel Assignment	Single intratracheal administration	3 × 10^6 cells per kg
							Normal saline without hUCB-MSCs
hCT-MSCs	NCT04255147	I	Cellular Therapy for Extreme Preterm Infants at Risk of Developing Bronchopulmonary Dysplasia	recruiting	Single Group	intravenous infusion	1 million cells/body kg; 3 million cells/body kg; 10 million cells/body kg
hUCB-MSCs	NCT01297205	I	Safety and Efficacy Evaluation of PNEUMOSTEM^®^ Treatment in Premature Infants With Bronchopulmonary Dysplasia	completed	Single Group	Single intratracheal administration	Dose A- 10 million cells per kg; Dose B- 20 million cells per kg
hUCB-MSCs	NCT01632475	I	Follow-Up Study of Safety and Efficacy of Pneumostem^®^ in Premature Infants With Bronchopulmonary Dysplasia	active, not recruiting	Single Group	Single intratracheal administration	Low Dose Group: 1.0 × 10^7 cells/kg; High Dose Group: 2.0 × 10^7 cells/kg
hUCB-MSCs	NCT04003857	II	Follow-up Study of Safety and Efficacy in Subjects Who Completed PNEUMOSTEM^®^ Phase II (MP-CR-012) Clinical Trial	recruiting	Parallel Assignment	Single intratracheal administration	1.0 × 10^7 cells/kg
							Saline without hUC-MSCs
hUCB-MSCs	NCT01828957	II	Efficacy and Safety Evaluation of Pneumostem^®^ Versus a Control Group for Treatment of BPD in Premature Infants	completed	Parallel Assignment	Single intratracheal administration	1.0 × 10^7 cells/kg
							Normal saline without hUCB-MSCs
hUCB-MSCs	NCT01897987	II	Follow-up Safety and Efficacy Evaluation on Subjects Who Completed PNEUMOSTEM^®^ Phase-II Clinical Trial	completed	Parallel Assignment	Single intratracheal administration	1.0 × 10^7 cells/kg
							Normal saline without hUCB-MSCs
hUCB-MSCs	NCT03392467	II	PNEUMOSTEM for the Prevention and Treatment of Severe BPD in Premature Infants	recruiting	Parallel Assignment	N/A	hUCB-MSCs
							Normal saline without hUCB-MSCs
hUCB-MSCs	NCT02023788	I	Long-term Safety and Efficacy Follow-up Study of PNEUMOSTEM^®^ in Patients Who Completed PNEUMOSTEM^®^ Phase-I Study	completed	Single Group	Single intratracheal administration	Low Dose Group: 1.0 × 10^7 cells/kg; High Dose Group: 2.0 × 10^7 cells/kg

hUC-MSCs, human umbilical cord mesenchymal stem cells; hCT-MSCs, human cord tissue mesenchymal stem cells; hUCB-MSCs, human umbilical cord blood-derived mesenchymal stem cells.

A case study recounted the treatment of an extremely premature baby suffering from severe BPD who received MSC therapy (1 × 10^7^ cells/kg/dose) both intratracheally and intravenously on the 78th day after birth. Before the treatment, the baby had been dependent on mechanical ventilation, initially with volume-targeted conventional therapy and later with high-frequency oscillatory ventilation. Despite receiving four doses of surfactant and undergoing two cycles of steroid therapy, the infant showed no improvement (Yilmaz et al., 2021). Following the MSC treatment, no deterioration in blood gas or indications of sepsis were noted, and there were no reported adverse events. The treatment was initiated after detecting chronic changes in the infant’s chest X-ray on the 78th day after birth. It yielded positive results of decreased emphysema and alveolar damage (Yilmaz et al., 2021). This encouraging case study indicates that MSC therapy may offer a promising therapeutic avenue for managing severe BPD in premature babies in the future.

## 6 Prospects and challenges

Mesenchymal stem cells (MSCs) have shown promising potential as a treatment for bronchopulmonary dysplasia (BPD), a chronic lung disease affecting premature infants. MSCs possess immunomodulatory and regenerative capacities that may mitigate lung injury and facilitate repair. However, several challenges must be addressed before MSC therapy can be widely adopted for BPD.

A significant hurdle is the inherent variability of MSCs depending on tissue source. MSCs derived from different donors and adult tissues exhibit differences in proliferative capacity, migratory abilities, cytokine secretion, and other functions relevant to BPD treatment. Standardizing and optimizing MSC-based therapies will require identifying the ideal MSC populations and culture methods tailored to BPD pathology. Factors influencing the localization, survival, and bioactivity of administered MSCs in the injured neonatal lung remain unclear.

Another concern is the potential influence of MSCs on cancer development. While MSCs appear less tumorigenic than other stem cells, their long-term safety requires further investigation, especially in vulnerable neonate populations. Animal studies have not detected tumor growth, but clinical evidence is still lacking.

It is imperative to note that MSCs are not intrinsically immune-privileged, and both autologous and allogeneic ways can stimulate anti-MSC immune responses, which can negatively impact their survival and efficacy. It is crucial to understand these complex dynamics to improve MSC-based therapies.

Additionally, guidelines are needed for identifying patients most likely to benefit from MSC treatment and the optimal administration timeline. Recent trials in ventilated preterm infants suggest efficacy, but more extensive studies are necessary to evaluate long-term outcomes and standardize cell production. Variations in manufacturing processes can alter MSC potency.

Future research should clarify mechanisms underlying MSC-mediated lung repair in BPD, such as paracrine signaling via EVs mitochondria through more giant vesicles (over 500 nm wide) or direct connections between cells. Strategies to enhance MSC engraftment and survival post-transplantation warrant exploration. As MSC therapy for BPD advances, international consensus on cell preparation, characterization, and delivery protocols will enable meaningful comparisons across clinical trials. A precision medicine approach adapting MSC therapy to each patient’s disease phenotype may prove optimal.

## 7 Conclusion

Bronchopulmonary dysplasia (BPD) is a chronic pulmonary disease that affects preterm infants, and its prevalence is increasing due to improved survival rates among this at-risk group. This condition arises from various factors, including maternal health, prenatal environment, and modern treatments for preterm babies, such as mechanical ventilation. Recent experimental studies have increased our understanding of the underlying pathogenetic mechanisms of BPD and revealed potential therapeutic targets. As BPD primarily manifests as a functional decline and reduction in lung stem cell populations, the prospect of replenishing and regenerating these cells could be crucial to its treatment. Recent clinical trials have validated the safety and effectiveness of MSC transplantation, with outcomes including a reduction in inflammation, mitochondrial oxidative stress, lung damage, and fibrosis. MSC-based therapy holds significant promise for promoting lung repair and regeneration in BPD. Realizing the full potential of MSCs will require systematic preclinical studies to elucidate mechanisms, optimize treatment protocols, and assess long-term safety. Well-designed clinical trials are needed to evaluate different MSC sources, combination therapies, and cell-free approaches. If critical challenges are addressed, MSCs could become an effective treatment for this debilitating developmental lung disease.
